# Breast Cancer in Pakistan: Alarming Situation of Breast Cancer in Near Future

**Published:** 2020-04

**Authors:** Muhammad ARIF, Mohsin JAVED, Hamid RAZA

**Affiliations:** Department of Chemistry, School of Science, University of Management and Technology, Lahore, Pakistan

## Dear Editor-in-Chief

Cancer is one type of diseases family, while breast cancer is a single disease which is the most common type of cancer in all over the world. Moreover, in the whole world, for women, it poses a common health risk (1). The breast cancer of Woman is in alarming situation in Pakistan long with all over the word. Its rate is increasing in every second (2). From the reported papers it is cleared that every 1 from 9^th^ women is susceptible to this health risk. The amount of this is present in mostly younger age females perhaps due to lack of self-confidence, feeling hesitation for checkup during breast test and lack of awareness (3). The other factors are marital status smoking, life style, diet, and positive family history, and environmental exposures (4). Mostly, Married females suffer from this disease (2). Breast cancer containing persons have various molecular, pathological and medical features which can directly effect on prognosis and treatment (5).

Statistical data has been reported from Shaukat Khanum Memorial Cancer Hospital & Research Center, Pakistan from January 1, 2018 to December 31, 2018 which was collected from Punjab, Gilgit-Baltistan & Kashmir, Khyber Pakhtunkhwa & F.A.T.A, Balochistan, Sindh, Federal Capital and 1270 breast cancer patients were reported during last year from which 1259 were female patients (6). The other reported study which was collected from Karachi, Pakistan, indicated that the rate of breast cancer is at the highest level among all Asian and Western countries except for Israel. In reported data from Karachi, 53.1% occurrence of breast cancer but in 1998–2002 it was 69.1% and in 2010–2012, it was 79.2% (7,8). Mostly the breast cancer is present in 40 plus aged female (4). The ratio of breast cancer is the most common than other types of cancer (8).

In conclusion, in near future, almost all female containing breast cancer in Pakistan as well as all over the world. New hospitals for cancer treatment must be built and the latest equipment’s and techniques are adopted to control this issue. Education about cancer especially breast cancer should be prevailed by media, books, magazines, newspapers etc and other sources in the world.
